# Role of Peak Nasal Inspiratory Flow for Measuring the Effectiveness of Surgery in Children with Adenoidal Hypertrophy

**DOI:** 10.7759/cureus.12378

**Published:** 2020-12-30

**Authors:** Abdurrahman B Cengiz, Hasan Deniz Tansuker, Cemal Ozyilmaz, Sinan Eroglu, Sahin Ogreden, Mehmet F Oktay

**Affiliations:** 1 Otolaryngology - Head and Neck Surgery, Bagcilar Training and Research Hospital, Istanbul, TUR; 2 Otolaryngology - Head and Neck Surgery, Bahcelievler State Hospital, Istanbul, TUR; 3 Otolaryngology - Head and Neck Surgery, Basaksehir Cam and Sakura State Hospital, Istanbul, TUR

**Keywords:** peak nasal inspiratory flow, adenoidectomy, simple visual analogue scale

## Abstract

Objective

Nasal airway obstruction in children is a frequent problem in otolaryngology practice. Adenoidal hypertrophy (AH) is the most common pathology in childhood that requires surgery. Nasal patency can be evaluated by subjective and objective methods. Unlike other methods, peak nasal inspiratory flow (PNIF) is portable and easy to perform. The need for patient compliance is the most important disadvantage of this method. We aimed to analyze the significance of PNIF for measuring the effectiveness of adenoidectomy as well as to compare PNIF with other subjective methods.

Methods

Two-hundred forty-five (245) patients aged between six and 11 years were evaluated. Seventy-seven (77) of them formed the study group and 168 formed the control group. Pre and post-surgery PNIF measurements, adenoid scores, and simple visual analog scale (sVAS) were recorded.

Results

The average PNIF value has significantly increased to 70.65 L/min from 33.02 L/min after adenoidectomy (p<0.01). The average PNIF value was 71.66 L/min in control subjects. High PNIF values were significantly correlated with low sVAS and adenoid scores postoperatively in the study group as compared with those of preoperative data (p<0.01).

Conclusions

PNIF has a satisfying correlation with nasal examination findings and other subjective methods to evaluate nasal obstruction and may provide unique and complementary information helpful for evaluating and improving the effects of adenoidectomy in children.

## Introduction

Nasal congestion is a common problem in pediatric otolaryngology practice. Nasal obstruction may be related to body position, age, the presence of infection or hypertrophy of the turbinates or lymphoid tissue (tonsils or adenoids), nasal polyps, allergies, or may result due to the adverse effect of some drugs [1]. Rhinoscopy is a reliable examination in order to decide the degree of nasal obstruction.

Chronic and recurrent allergic and infectious events cause lymphoid overgrowth in the nasopharynx. Adenoidal hypertrophy (AH) obstructs the nasal airflow passage at the choana area and causes one of the most frequent surgery indications in the pediatric population [2]. AH is related to other diseases seen in daily practice such as recurrent otitis media, chronic rhinosinusitis, and obstructive sleep apnoea [3].

Objective measures for the initial evaluation of the severity of the nasal airflow have been performed for years by otolaryngologists [4]. The methods currently available for this purpose are acoustic rhinometry (AR), rhinomanometry (RM), and peak nasal inspiratory flow (PNIF). RM and AR are complex and, therefore, infrequently used in clinical practice. They require more expensive equipment than PNIF with nearly the same ability to estimate nasal airflow, which is an objective, non-specific measurement for nasal obstruction and response to treatment regardless of the etiology.

Recently, the visual analog scale (VAS) enables an uncomplicated, rapid, and reproducible evaluation of nasal symptom severity and has been used for AH before [5].

We aimed to investigate the role of PNIF to evaluate the efficiency of adenoidectomy as well as to compare PNIF with physical examination and simple visual analog scale (sVAS) scores.

## Materials and methods

Children referred for adenoidectomy to the Bagcilar Training and Research Hospital, Istanbul, Turkey, a tertiary rhinology referral center, from January 2019 and 2020, were consecutively treated. The study group included 77 patients (42 girls and 35 boys) aged between six and 11 who underwent only adenoidectomy for adenoidal vegetation. Children younger than five years old were excluded, as they could not comply with the instructions. In order to establish the normal parameters of PNIF for healthy children, the control group included 168 individuals without upper airway obstruction. We used the Youlten Flowmeter (Clement Clarke International, Essex, UK) to measure the forced nasal inspirium.

Inclusion and exclusion criteria

Inclusion Criteria

Patients who underwent elective adenoidectomy between six and 11 years and the healthy control group were included in the study. The patients who were admitted to our clinic with snoring and chronic mouth breathing complaints and having AH causing nasal obstruction by closing the choanae, with a normal tonsilla palatina and no turbinate hypertrophy and no septum deviation in the examination. Fiberoptic nasopharyngoscopy and lateral graphy were used for the adenoid examination.

Exclusion Criteria

Children found to have sinusitis, deviated septum, nasal polyps, upper respiratory tract infection, turbinate hypertrophy, upper tract surgery or active medication history, or cleft palate; those unable to perform the maneuver to obtain PNIF; and patients with any pulmonary and cardiac chronic disease or surgery history that may affect inspiration capacity were excluded. Children with tonsillar hypertrophy were also excluded from the study.

Surgery

The surgical procedure was performed under general anesthesia by the same surgent using classic curettage technic removed the adenoids through the mouth. The patients were discharged on the day of the operation.

Measurements

PNIF, expressed in L/min, is defined as the maximum breath obtained at once through the nose. Before measuring PNIF, subjects performed a usual nasal hygiene routine, mildly blowing their noses to clear up secretion. The facial mask was placed while standing. The participants were instructed to do a nasal inspiration with their mouths closed and from the residual volume. At least, three measurements were done and the maximum value recorded was considered for analysis until reaching their total pulmonary capacity [6]. The PNIF measurement was performed in patients preoperatively and three months after the operation.

The nasal examination was performed with a rigid endoscope after topical anesthesia with procaine (NTcain; Assoc Drugs, Turkey). AH was scored as four grades, according to the distance between the adenoid tissue and vomer as reported by a previous study [7]. Grade 1 was described as distance more than 1 cm, grade 2 between 0.5 and 1 cm, grade 3 distance less than 0.5cm, and total obstruction was noted as grade 4.

sVAS, which was described as a modified survey in the literature [1], was used to determine the severity of obstruction from the patient's perspective, including open mouth sleep and snoring, which starts from 1 (no obstruction) to 4 (severe obstruction). The survey was managed preoperatively and postoperatively three months afterward.

Statistical analysis

The analysis of variance (ANOVA) test and the Kruskal-Wallis test for variables with normal and non-normal distribution were performed. The student's t-test was used for normally distributed parameters and the Mann-Whitney U test was used for non-normal distributed parameters. The paired-sample t-test was used for the comparison of preoperative and postoperative data of normal distribution, and the Wilcoxon signed-rank test was used for a comparison of non-normally distributed parameters. P <0.05 was considered significant. The Statistical Package for the Social Sciences (SPSS) 20.0 (IBM Corp., Armonk, NY) was used in the analysis.

## Results

This prospective study included 245 patients aged between six and 11 years (mean age was 7.97±1.81 years). One-hundred nineteen girls (48.5%) and 126 boys (51.5%), of whom 77 underwent adenoidectomy and of whom 168 were healthy volunteers. Thirty-six boys (46.7%) and 41 (53.3%) girls were operated on. The average age of the operated group was 8.06±1.71. No difference was found between the study and control groups regarding age and gender (p=0.786).

The mean values of PNIF and SD for boys and girls of the study and control groups, according to their age are shown in Table 1. The control group had a statistically significant higher PNIF value as compared to the preoperative PNIF value of the study group (p<0.01) There was no difference between the study and control groups regarding weight and height (p>0.05). Significant increases in weight, height, and PNIF values for boys and girls were observed in relation to age (p<0.01).

**Table 1:** Mean, standard deviation (sd), for weight, height, and PNIF (L/min) for boys (b) and girls (g) according to their age (per year) PNIF: peak nasal inspiratory flow

		STUDY GROUP (PREOPERATIVE)			CONTROL GROUP		
		Weight (kg)	Height (cm)	PNIF value(L/min)	Weight (kg)	Height (cm)	PNIF value^ *^(L/min)
Age	Gender	Mean±SD	Mean±SD	Mean±SD	Mean±SD	Mean±SD	Mean±SD
6	B	19,603,68	115,55,25	22,50±9,20	19,54±3,21	116,23±5,71	56,73±16,47
	G	18,6±62,95	114±6,91	21,667,07	18,25±2,79	112,23±4,35	48,12±12,88
7	B	19,8±54,33	121,22±7,41	27,14±9,06	20,25±3,39	122,77±6,18	68,88±16,26
	G	20,37±3,29	120,37±5,39	24,37±9,03	18,36±3,11	115,96±5,77	50,55±14.59
8	B	23,85±3,13	123,28±6,21	30±7,63	23,07±3,23	125,61±6,15	69,61±15,67
	G	21,50±2,58	124,33±7,42	24,37±9,03	20,12±3,01	116,65±4,11	55,98±13.22
9	B	27±6,04	129,60±3,73	35±7,90	27,54±4,45	129,72±4,92	77,27±17,09
	G	27,5±3,56	123,50±5,08	35,83±12,81	23,15±3,21	122,79±4.20	65,32±16,35
10	B	34,40±4,44	134±5,19	46±11,40	33,50±3,81	133,80±4,37	82,00±15,59
	G	32,60±3,50	132,20±4,54	43±9,74	29,52±3,26	124,69±4.88	70,33±14,45
11	B	34,20±7,42	139,80±5,50	58,1±7,55	33,44±6,47	141,22±5,21	100,00±6,41
	G	32,25±6,94	135,45±6,87	57,50±11,21	30,89±5,12	135,35±5,71	82,42±11,16
	Average	24,6±36,95	124,761±0,00	33,44±14,40	24,44±6,76	125,62±9,64	71,66±20,02
	TOTAL	N=77			N=168		

**Figure 1:**
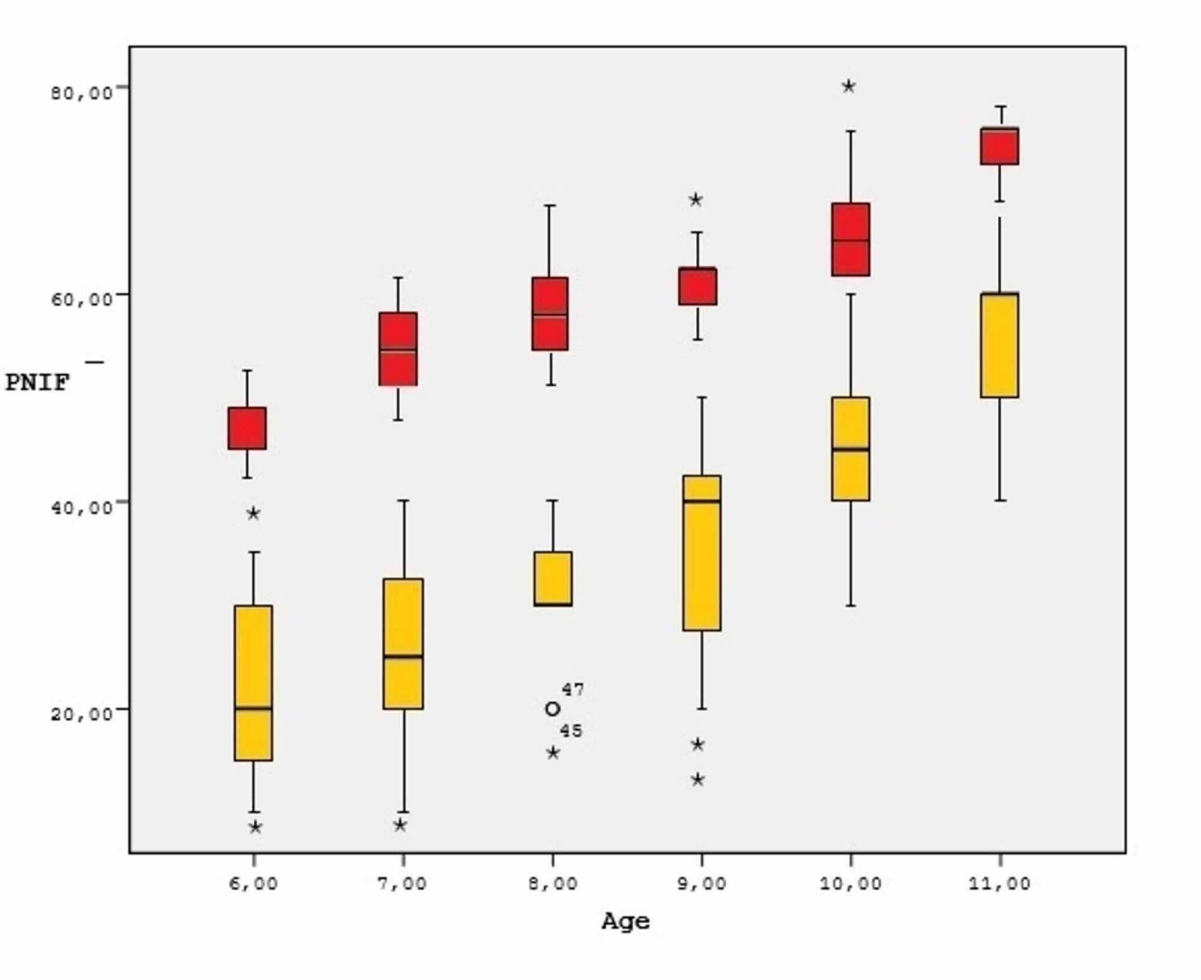
Mean and 95% confidence interval (CI) for PNIF according to age (years) Yellow: preoperative PNIF; Red: postoperative PNIF PNIF: peak nasal inspiratory flow

PNIF and sVAS degrees between study and control groups were shown in Table 2. The postoperative PNIF value of the study group was not significantly different as compared to the control group (p>0.05). Postoperative PNIF measurements of the study group were found as being improved after the operation. Table 3 (p<0.01). The postoperative sVAS and adenoid scores of the study group were found significantly lower than preoperative data (p<0.01.) PNIF values for each percentile for patients in relation to their age are shown in Table 1. A significant increase of PNIF values for boys and girls was observed in relation to age increase. Figure 1 shows the mean and 95% confidence interval (CI) for PNIF according to age (years).

**Table 2:** Comparison of preoperative and postoperative PNIF and VAS scores of the study group with PNIF and VAS scores of the control group PNIF: peak nasal inspiratory flow; VAS: visual analog scale; sVAS: simple visual analog scale

	Study Group	Control Group	
	Mean±SD	Mean±SD	P
Preoperative PNIF value^a^	33.44±14.40	71.66±20.02	0.001*
Postoperative PNIF value	72.46±20.04	71.66±20.02	0.772
Preoperative postoperative^b^ P	0.001*		
Preoperative sVAS value^c^	3.48±0.59	1.22±0.42	0.001*
Postoperative sVAS value	1.33±0.47	1.22±0.42	0.056
Preoperative postoperative^d^ P	0.001*		
*P < 0.001 ^a^Student's t-test. ^b^Wilcoxon signed-rank test. ^c^Mann-Whitney U test. ^d^Paired samples t-test			

**Table 3:** Comparison of preoperative and postoperative adenoid score and PNIF and sVAS values of the study group PNIF: peak nasal inspiratory flow; sVAS: simple visual analog scale

		Adenoid Score		PNIF value (L/min)		sVAS value		
Age	Gender	Mean±SD		Mean±SD		Mean±SD		
		Preop	Postop	Preop	Postop	Preop	Postop	
6(n=19)	B=10	3,30±0,67	1,10±0,31	22,50±9,20	60±14,90	3,10±0,70	1,20±0,42	
	G=9	3,22±0,66	1,22±0,44	21,667,07	54,44±17,21	3,77±0,44	1,44±0,52	
	N=19	3,26±0,65	1,15±0,37	22,10±8,04	57,36±15,84	3,42 ±0,69	1,31 ±0,47	
7(n=15)	B=7	3,28±0,75	1,00	27,14±9,06	77,85±14,87	3,71±0,48	1,28±0,48	
	G=8	3,25±0,88	1,37±0,51	24,37±9,03	58,75±20,48	3,12±0,64	1,50±0,53	
	N=17	3,26±0,79	1,20±0,41	25,66±8,83	67,66±17,81	3,40 ±0,63	1,40 ±0,50	
8(n=13)	B=7	3±0,81	1,28±0,48	30±7,63	61,42±17,49	3±0,57	1,42±0,53	
	G=6	2,83±0,75	1	24,37±9,03	79,16±6,64	3,83±0,40	1,33±0,51	
	N=13	2,92±0,75	1,15±0,37	31,92±6,62	69,61±16,02	3,38 ±0,65	1,38 ±0,50	
9(n=11)	B=5	3,40±0,89	1,10±0,02	35±7,90	77,00±21,09	3,20±0,44	1,40±0,54	
	G=6	3,50±0,83	1,10±0,31	35,83±12,81	77,50±16,04	3,66±0,51	1,50±0,54	
	N=11	3,45±0,82	1,09±0,30	35,45±10,35	77,27±17,51	3,45±0,52	1,45 ±0,52	
10(n=10)	B=5	3±0,4	1,15±0,37	46±11,40	88±8,03	3,60±0,54	1,42±0,53	
	G=5	3,20,0,83	1,20±0,37	43±9,74	76±20,43	3,80±0,44	1,30±0,46	
	N=10	3,10±0,73	1,10±0,31	44,50±10,02	82,00±16,02	3,70 ±0,48	1,10 ±0,31	
11(n=9)	B=5	3±0,81	1,10±0,33	58,1±7,55	99±7,41	3,66±0,54	1,25±0,50	
	G=4	3±0,86	1,12±0,33	57,50±11,21	101,2±5,638	3,75±0,50	1,33±0,50	
	N=9	3±0,86	1,10±0,31	57,78±11,21	100±6,61	3,66 ±0,5	1,33 ±0,5	
Total=77	B=39	3,18±0,75	1,12±0,33	33,44±14,40	72,46±20,04	3,48 ±0,59	1,33 ±0,47	
	G=38	3,17±0,75	1,07±0,26	33,74±14,34	74,23±18,90	3,33±0,62	1,30±0,46	
	N=77	3,18±0,75	1,15±0,36	33,02±14,63	70,65±21,25	3,63±0,54	1,36±0,48	

The sVAS data of the study group preoperatively were significantly higher as compared to the control group (Table 1) (P<0.01). The postoperative sVAS scores of the study group and the sVAS scores of the healthy control group were similar (p>0.05).

The operated group had significantly lower postoperative sVAS scores as compared with the preoperative sVAS scores (Table 3) (p<0.01). The sVAS and adenoid scores demonstrated a negative significant correlation with PNIF (r=-.664, p=0.001 for the former and r = -.752, p=0.001 for the latter).

There were eight patients (four boys, four girls) with postoperatively high sVAS scores. PNIF levels were also found lower as compared to the other operated patients and the healthy group. The correlation between PNIF and sVAS values seems important; however, it is statistically not significant (p=0.612).

## Discussion

Nasal obstruction is a frequent complaint in children; in any case, its evaluation is barely reliable [8]. The etiology of AH has not been completely understood, however, it could be commonly thought that persistent, serious, and repetitive inflammatory conditions developing around the adenoid tissue are critical at this point [9].

The objective evaluation of nasal patency may add relevant information about the nasal function and be valuable for the management of upper airway disorders. Such convenience happens mainly among children in which objective measurements are even more adequate because of the particularity of the subjective information that is often provided by parents.

PNIF is a simple, efficiently performed measurement and may be correlated to the clinical evaluation of patients with nasal blockage. It has been used for years as a good approach for estimating nasal obstruction because it can be effortlessly performed and construed [10]. Possible reasons for the inaccuracy of PNIF include random and technical operating errors such as measurements with loose face masks or inadequately closed mouth.

A few studies have been detailed on the viability of PNIF. RM, AR, and VAS have been compared to PNIF regarding the evaluation of nasal obstruction [11-13]. Recently, Ottaviano et al. compared RM and PNIF in healthy and obstructed noses and reported that each test has significant value to discriminate disease [14]. Nonetheless, RM has been considered to be preferable for assessing each nasal cavity independently [15]. RM is not as significant as AH because the disease affects both sides.

It was reported that endoscopic nasal examination has reliability for the identification of AH; however, anterior rhinoscopy examination is unable to assess the obstruction level in small or disoriented children. Lateral cephalometry is a noninvasive test to accurately assess the grade of the AH in patients [16]. However, lateral graphy is not sufficient for clinicians in some cases, and we need another objective decision-making test for surgical treatment.

It is essential to have knowledge of the standard values of PNIF. In children and the adolescent population, there are studies that established curves with normal variation, without nasal problems, and provide formulas and charts correlating height, age, and gender with PNIF [17-18]. Papachristou et al. studied 3170 Greek children and adolescents from six to 11 years old and presented reference curves of PNIF for boys and girls [19]. Mean values ​​ranged from 80 to 130 L/min. In another study, the equations of the final regression model were shown as the following formula, with reference values of PNIF for boys and girls, respectively (= 0.7x Height (cm)+11,2 and = 0.7x Height (cm). It was seen that boys, in all age groups, had higher PNIF values as compared to girls) [18]. Our study showed that for both boys and girls, the measured mean values of PNIF increase with age and are higher in boys as found in other studies [17]. We observed lower PNIF values in our patients as compared with healthy children, which points out that PNIF may be valuable for the evaluation of nasal congestion.

Ozkul et al. found that pre and postoperative PNIF and VAS data are correlated in adult patients that had a septoplasty and appear to be a really viable method within the assessment of nasal obstruction and in choosing the operation [13]. We observed that PNIF measures significantly improved at six months after adenoidectomy. In another study, a similar significant negative relationship was found between PNIF and the Nasal Obstruction Symptom Evaluation (NOSE) scale of patients undergoing septorhinoplasty [20]. Sandhu et al. [21] showed significant improvements in the PNIF and VAS scores of patients undergoing partial laser turbinectomy in their study. In our study, we found that endoscopic examination and obstruction symptoms (sVAS) have a significantly negative correlation with PNIF.

Ciprandi et al. compared the usage of sVAS with the endoscopic examination and reported that it may allow objectifying the efficiency of surgery with good reliability in the absence of rhinomanometry [1]. In our study, sVAS was found to have a significant correlation with the adenoid mass and tends to decrease with adenoidectomy.

To our knowledge, this is the first study in which isolated adenoidectomy was evaluated by PNIF. We observed that both sVAS scores and PNIF had a clinically significant recovery from the baseline following adenoidectomy. PNIF can, in fact, be helpful for evaluating and understanding the efficiency of surgical techniques and may be beneficial to assess objective nasal passage airflow changes of patients following surgery.

## Conclusions

The results of the present study suggest that pediatric patients need to be evaluated with both objective and subjective techniques. Nasal endoscopy is the most accurate method. In the parent's (or children's) perception, sVAS has a good relationship with nasal endoscopy findings. PNIF is a portable, inexpensive, non-invasive, and very effective method that may help clinicians in current practice to supply broad data on nasal disfunction. PNIF is a useful method to assess effectiveness as well as the decision to operate.
